# The EvalUation of goal-diRected activities to prOmote well-beIng and heAlth (EUROIA) scale: Psychometric evaluation with Confirmatory Factor Analysis

**DOI:** 10.1371/journal.pone.0299854

**Published:** 2024-03-14

**Authors:** Troy Francis, Rachel G. Peiris, Aleksandra Stanimirovic, Nicolette Stogios, Fatima Syed, Valeria E. Rac, Robert P. Nolan

**Affiliations:** 1 Program for Health System and Technology Evaluation, Ted Rogers Centre for Heart Research, Toronto General Hospital Research Institute, University Health Network, Toronto, Ontario, Canada; 2 Institute of Health Policy, Management and Evaluation, Dalla Lana School of Public Health, University of Toronto, Toronto, Ontario, Canada; 3 Cardiac eHealth, Peter Munk Cardiac Centre, University Health Network, Toronto, Ontario, Canada; 4 Institute of Medical Science, University of Toronto Faculty of Medicine, Toronto, Ontario, Canada; 5 Department of Psychiatry, University of Toronto, Toronto, Ontario, Canada; Zhejiang Shuren University, CHINA

## Abstract

**Objectives:**

While state-based models of health-related quality of life (HRQL) are well-established in providing clinically relevant descriptions of HRQL status, they do not provide information on how to maintain or improve HRQL. The EvalUation of goal-diRected activities to prOmote well-beIng and heAlth (EUROIA), rooted in a novel process-based model of HRQL, measures goal-directed activities that are self-reported to promote HRQL as part of an individual’s process of adapting to dynamic changes in health status. Our objectives were to condense and summarize the psychometric properties of the EUROIA by (i) defining and confirming its factor structure, (ii) evaluating its construct validity, and (iii) examining its internal consistency.

**Methods:**

Principal component analysis was performed on the 18-item EUROIA to explore the underlying factor structure and condense the scale. Confirmatory factor analysis was conducted on the revised 14-item, 4-factor structure EUROIA instrument to evaluate the model fit. Data was obtained from adult participants with a diagnosis of chronic heart failure or advanced chronic kidney disease from 3 hospitals in Toronto, Canada.

**Results:**

The revised 14-item EUROIA demonstrated 4 dimensions–Social Affiliation, fulfillment of Social Roles and Responsibilities, Self-Affirmation, and Eudaimonic Well-being–with a Cronbach’s alpha of 0.83, representing good internal consistency. Our confirmatory factor analysis final model achieved good overall model fit: (*χ*^2^ / df = 1.80; Tucker-Lewis index = 0.90; comparative fit index = 0.93; standardized root-mean-square residual = 0.06; root-mean-square error of approximation = 0.06). All items exhibited a factor loading greater than λ > 0.4 and *p* < 0.001.

**Conclusion:**

The EUROIA holds clinical potential in its ability to provide informed feedback to patients on how they might maintain or modify their use of goal-directed activities to maintain and optimize perceived well-being.

## Introduction

Health-related quality of life (HRQL) is defined as the appraisal of the impact of a medical condition on one’s physical and mental health and well-being [1]. It is advocated as a patient-reported outcome for clinical research, health services research, and health care by international medical associations [2–5]. HRQL is commonly assessed using multidimensional profiles that are based on self-ratings of physical symptoms, activities of daily living, and cognitive, emotional, and social functioning at a specified timepoint [6, 7]. Each profile is scored and interpreted in reference to an idealized HRQL state in which life is unimpeded by negative emotional or physical symptoms or functional impairments. Items that comprise these state-based HRQL profiles have been shown to reflect three philosophical models of well-being; pleasure and life satisfaction that outweigh symptoms of emotional or physical distress or discomfort (hedonic well-being), maintenance of individual and social activities associated with personal flourishing (eudaimonic well-being), and satisfaction versus frustration of one’s desires [8]. Several generic and disease-specific psychometric assessments of HRQL have established validity and reliability [9, 10]. along with demonstrated prognostic value due to their association with clinical outcomes [11].

### EvalUation of goal-diRected activities to prOmote well-beIng and heAlth (EUROIA)

The term EUROIA is derived from the ancient Greek term for “good flow of life” (*euroia biou*), which was central to a revision of Aristotle’s concept of eudaimonia as taught in ancient Stoic philosophy [12–14]. *Euroia biou* was presented as a therapeutic guide for accepting and responding to all life events–those associated with the development of personal growth and flourishing, as well as those that evoked distress, pain, or suffering. It asserted that well-being was ultimately experienced as a by-product of our balanced effort to change realities that are under our control, and to live in agreement with, and contribute to, the rational order in events which we perceive as being outside of our control. “Good flow in life” can be viewed as a translation of having a life characterized by biopsychosocial wellbeing–being able to respond to life’s challenges and adversities in a way that promotes psychological health and flourishing. This philosophy pertains to our relationships with family and significant others, our role as citizens of society-at-large, and our status as members of the natural world and, ultimately, the cosmos [12, 15].

State-based models provide valuable information pertaining to HRQL status and the risk of future clinical events, they provide insufficient information for maintaining or improving an individual’s HRQL. To this end, we propose that a state-based model may be used in tandem with a process-based model to reflect and inform the self-regulatory process of adapting to dynamic changes in health status [8, 16]. We developed items that comprise the EUROIA based on 3 sources. First, findings from qualitative studies of patients with chronic heart failure indicate that self-reported HRQL reflects an adaptive process whereby activities for living well are initiated or maintained to support intrinsic personal priorities or life goals [8]. We previously described these goal-directed themes as including the following: (i) sustaining personal growth, (ii) participating in life events that are perceived as being meaningful, (iii) sharing time with a loved one, family, or friends, (iv) fulfilling social roles and responsibilities in which one is emotionally invested, or (iv) maintaining physical health and emotional well-being [8]. Second, extensive evidence from studies in response-shift theory indicate that self-reported HRQL changes over time as patients (i) recalibrate how they use rating scales for HRQL items, (ii) reprioritize the relative importance of different components of HRQL that match salient features in their evolving appraisal of HRQL, and (iii) reconceptualize the overall HRQL construct in keeping with novel experiences and insights that shape their evolving view of what ultimately constitutes a good life [17]. Third, the EUROIA is distinct from conventional assessments whereby the HRQL state incorporates themes of well-being that focus on individual self-interest–i.e., hedonic or eudaimonic well-being or desire-satisfaction. In contrast, the EUROIA focuses on goal-directed activities that include the individual’s effort to (i) provide valued support or assistance to help significant others stay safe and secure, or (ii) feel part of a greater purpose in life, or (iii) feel connected to nature or the natural world. In sum, the EUROIA differs from current assessments of an individual’s functional HRQL state because it reflects a process-based model of HRQL. The EUROIA yields information about the individual’s engagement in goal-directed activities for living well, which are associated with their effort to maintain a sense of personal agency or autonomy as they adapt to dynamic changes in their health status or bio-psycho-social environment.

Theoretical models have guided the development of psychometric instruments targeting the umbrella terms *HRQL* and *well-being*. For the EUROIA, several models played a key role in informing its development. Items related to spending time with a significant other or with an individual with whom one is emotionally intimate were guided by social affiliation, a major determinant of health as it predicts mental and physical health, health behaviour, and mortality risk [18]. Social affiliation also enhances well-being by reducing loneliness [19] and increasing feelings of satisfaction [19, 20]. Another aspect of well-being identified by goal-directed activities in the EUROIA pertains to fulfilling social roles and responsibilities in relation to significant others. A growing body of work by Prilleltensky and colleagues [21–26] points to the importance of collective fairness, which balances the need to feel valued by one’s community or social group with the ability to make contributions to this group or community that are deemed valuable. In addition, qualitative findings indicate that activities which affirm one’s sense of health and well-being take on existential significance when self-reported by individuals diagnosed with a chronic medical condition that is associated with premature morbidity and mortality [8]. Goal-directed activities in the EUROIA’s self-affirmation theme included meditation, relaxation, and physical exercise. Evidence supporting the association between these self-affirming activities and self-ratings of well-being are based on mediators such as positive mood [27–29], optimism [27], emotion-focused coping [30], and self-efficacy, which promote engagement in self-care behaviour [27]. The dimension of eudamonic well-being is commonly identified in self-ratings of personal flourishing where an individual reports an ability to engage in activities that promote personal growth and mastery in skill or knowledge [31]. The EUROIA identified goal-directed activities with a eudamonic well-being theme. There is extensive evidence that links markers of eudaimonic well-being to longitudinal indices of subjective health and decreased risk for chronic medical conditions and functional impairment [32].

The aim of the present study was to condense and summarize the psychometric properties of the EUROIA [16]. We (i) defined and confirm the factor structure of the EUROIA instrument, (ii) evaluated its construct validity, and (iii) examined its internal consistency.

## Methods

### EUROIA

Items in the EUROIA have been reported to reflect activities that evoke or sustain well-being along the following dimensions: Social Affiliation (SOC-AFF), fulfillment of Social Roles and Responsibilities (SOC-RR), Self-Affirmation (SLF-AFF), and Eudaimonic Well-being (EUD-WB). SOC-AFF is comprised of behaviors such as participation in social activities, maintaining close or intimate relationships, and helping family or friends, or others who are perceived to be in need. SOC-RR includes activities aimed at maintaining social roles and responsibilities in relation to significant others and being productive in work settings. SLF-AFF pertains to activities that promote feelings of health, attractiveness, emotional calm, and physical well-being via physical activity or exercise in the context of managing a chronic progressive medical condition. EUD-WB consists of behaviors that promote individual flourishing, personal growth, engagement with the natural world, and life satisfaction [33, 34].

Principal component analysis (PCA) was performed on the EUROIA at different stages of its development [16]. Given our previous report of a 4-factor structure for the original version of the EUROIA, we used PCA to explore the underlying structure of a revised 18-item questionnaire where the main purpose was to condense the scale. Confirmatory factor analysis (CFA) was performed on the condensed EUROIA instrument whereby the objective was to evaluate the model fit. EUROIA items were developed to be gender-neutral and easily interpretable. It has a Flesch Kinkaid reading level [35] of 7.5. A 2-week timeframe was selected to ensure realistic reports of the frequency of goal-directed activities that were initiated or maintained to promote a sense of “living well”. Early research showed that a 2-week timeframe was effective in assessing patient-reported behavioral outcomes [36]. Likert-type scales measured activity frequency: *0 = not at all*, *1 = one day a week*, *2 = two to three days a week*, *3 = four or more days a week*. Reliability of the EUROIA was a Cronbach’s alpha (*α*) of 0.77 at baseline and 0.82 at 12 months [16].

### Data source and study population

This investigation was based on the baseline assessment for 2 projects that were conducted in parallel, and which used the same digital counseling program to promote HRQL: the **O**pen access **D**igital communit**Y** promoting **S**elf-care, peer **S**upport, and h**E**alth lit**E**racy–a **V**irtual **C**ommunity promoting mental **H**ealth, self-c**A**re, and peer suppor**T** (ODYSSEE-vCHAT). The recruitment period for this study began October 25^th^, 2021 and ended on January 16^th^, 2023. Baseline assessment data were obtained from (i) a single group, open label, pre-post study [37] and (ii) a double-arm, parallel group, randomized controlled trial [38]. The sample estimate for the ODYSSEE-vCHAT study accounted for changes in the Mental Component Score from the 36-Item Short Form questionnaire over a period of 3 to 12 months, while the sample estimate for the ODYSSEE-vCHAT trial was based on change over 12 months in a composite index of all-cause mortality and hospitalization. The sample size estimate for the trial was *N* = 162 and the sample estimate for the study was *N* = 188 and included oversampling, with both the study and trial adjusted for a potential 12-month withdrawal or attrition rate of 14.7%, type 1 error of 5%, and a power of 80% [39]. Participants met the following inclusion criteria [37]: age 18 years or older, diagnosis of chronic heart failure (CHF; NYHA Class II to IV for at least 3 months prior to enrolment) or advanced chronic kidney disease (CKD; greater than 10% risk of requiring dialysis within 2 years or end-stage renal disease receiving dialysis). Participants were also required to be proficient in English, have access to a computer or tablet and a personal email address, and provide written informed consent. Exclusion criteria consisted of advanced surgical interventions within 3 months of enrolment, severe comorbidities that would impede participation (e.g., diagnosis of current alcohol or drug addiction, or severe depression), and a life expectancy of less than 1 year. We used automated coding algorithms published by each questionnaire and double-checked the results to minimize human error and reduce potential bias in data collection. To prevent non-response bias, individuals were required to complete a pre-established number of questions before scoring their responses. No financial incentives were offered. Participants were referred from participating clinics at the University Health Network (UHN; primary site), Mount Sinai Hospital, and Sunnybrook Hospital in Toronto, Canada [37, 38]. Assessment data for the present investigation were obtained from baseline assessments.

### Ethics

The ODYSSEE-vCHAT study [37] and trial [38] were conducted according to principles of the Declaration of Helsinki [40]. All procedures were reviewed and approved by the ethics board of each participating site (UHN: 20–5960 and 20–6005; Sunnybrook: 5117 and 3787; Mount Sinai: 21-022-E and 21-0057-E).

### Statistical analysis

Descriptive statistics provided an overview of baseline demographics, clinical characteristics, and HRQL of the patient sample. Continuous variables were described using means and standard deviations, and categorical variables were described using counts and percentages. Comparisons of EUROIA scores across men and women were analyzed using Linear Model ANOVA’s for continuous variables. Analyses were performed using R software v4.1.0 [41], psych [42] and lavaan packages [43]. To construct the measurement model in the CFA, at least 2 items are needed to estimate each dimension. Therefore, only multiple-item dimensions were included [16]. Most EUROIA items had less than 3% missing responses; therefore, multiple imputations were not necessary. Only raw scores and complete cases were used in the CFA.

#### PCA

PCAs with promax (oblique) rotations determined the factor structure of the EUROIA. Oblimin rotation was employed because dimensions were conceptualized as interrelated aspects of HRQL. Criteria for allocating items to principal components and establishing the component structure of the questionnaire were as follows: Eigenvalues > 1; examination of scree plots; factor loadings of coefficients ≥ 0.40 for individual items in the rotated PCA. Items were selected with the intention of reducing redundancy in the instrument. Deletion of items from the 18-item EUROIA was performed in several steps using *α*, factor loadings, and theoretical considerations based on a team member with clinical expertise. Internal consistency of the EUROIA was assessed using *α*. Item analyses included an assessment of internal reliability with item decisions. α was interpreted as follows: ≥ 0.7 is acceptable; ≥ 0.8 is good; *α* ≥ 0.9 is excellent [44, 45].

#### CFA

Mardia’s test for normality and quantile-quantile plots determined whether data were multivariate normally distributed [46] and suitable for CFA. The data suggested that item-level distributions violated the assumption of normality. CFA was then performed on the reduced EUROIA instrument using “MLM” as our estimator, i.e., a maximum likelihood estimation with robust standard errors and a Satorra-Bentler (SB) scaled test statistic [47, 48]. SB scaled *χ*^2^ statistics were used if high kurtosis statistics suggested that the items were not normally distributed, a non-significant SB *χ*^2^, where *p* > 0.05, is desired [47]. In the baseline factor model, the factors were allowed to correlate freely, the residuals were uncorrelated, and a standardized solution was obtained. We evaluated global model fit using:

- Root mean square error of approximation (RMSEA), which includes an adjustment whereby more complex models with greater degrees of freedom are penalized [47]. Cut-off values for fit are < 0.08 (adequate) and < 0.05 (good), with an upper limit of the 90% confidence interval of < 0.08 [49, 50].
- Standardized root mean square residual (SRMR), which is the mean absolute residual correlation whereby < 0.08 and < 0.06 indicate acceptable and good fit, respectively [49, 50].
- Comparative fit index (CFI) and Tucker-Lewis Index (TLI), which indicate improvement in fit compared to the baseline exact fit (*χ*^2^) model. > 0.90 is acceptable and > 0.95 is good [49, 50].
- An adjusted test statistic of SB *χ*^2^ or degrees of freedom (df) < 2, which indicates a good fit [51].
- Models that were compared using *χ*^2^ likelihood ratio tests. A lower chi-squared value indicated a better fit. Our team member with clinical expertise in behavioral health reviewed the content validity and clinical meaningfulness of retained dimensions.
- R^2^, which represented the proportion of variance in each item response explained by the factor (> 0.10) [52].

For models with poor global model fit, residuals and modification indices were inspected to identify local areas of strain. Correlated item residuals revealed a common variance unaccounted for by the initial factor structure, which occurred when item content overlapped, leading to suboptimal model fit [53, 54]. A modification index approximated the degree that a model’s *χ*^2^ statistic would decrease if a given fixed parameter became freely estimated [52]. A model with areas of local strain would consist of item pairs in the same dimension with a high modification indices (> 25.0) [55]. In the case of overlapping item content, we re-specified models to correlate item residuals and re-assessed residuals and modification indices. The independent clusters model of CFA estimated composite reliability (coefficient omega) to determine internal consistency of the EUROIA instrument [56].

## Results

Table 1 outlines demographic and clinical characteristics of participants (*N* = 275). The mean age was 56.6 years (range: 20.0 to 91.0, SD = 16.0). More participants self-identified as men (59.6%) than women (40.4%). Participants were diagnosed with CHF (50.5%) or CKD (49.5%).

**Table 1:** Demographic and clinical characteristics.

Demographic Characteristics	All Patients (*N* = 275)
**Highest level of education completed**	***n* (%)**
Secondary school	33 (12.0)
College or undergraduate	178 (65.0)
Graduate or post-graduate	63 (23.0)
**Household income (CAD)**	***n* (%)**
< 20 000	21 (8.0)
20 000 to 39 900	31 (11.8)
40 000 to 59 000	37 (14.1)
60 000 to 79 900	31 (11.8)
80 000 to 99 900	26 (9.9)
≥ 100 000	77 (29.3)
Prefer not to say	40 (15.2)
**Household status**	**n (%)**
Single and living alone	55 (20.8)
Single and living with others	53 (20.1)
Living with your partner	156 (59.1)
**Ethno-racial identity**	***n* (%)**
White	160 (56.7)
Black	24 (8.5)
Middle Eastern or North African	10 (3.5)
East Asian	17 (6.0)
Southeast Asian	16 (5.7)
South Asian	32 (11.3)
Hispanic	3 (1.1)
Indigenous	3 (1.1)
Other	17 (6.0)

Canadian dollars, CAD

### 14-item EUROIA

The final version of the EUROIA consisted of 14 items with four dimensions (factors): SOC-AFF, SOC-RR, SLF-AFF, and EUD-WB. Four items were removed from the initial instrument due to redundancies in the scale based on cross factor loadings and theoretical considerations. Bartlett’s test of sphericity was 906.4, *p* < 0.001, which determined that the EUROIA variables deviated significantly from an identity matrix. Similarly, the Kaiser-Meyer-Olkin test was 0.81, indicating that there was a sufficient proportion of variance among the EUROIA items that was attributable to common variance. Cronbach’s *α* for the 14-item EUROIA was 0.83, which represents good internal consistency. Total variance explained factor loadings (59.1%), and *α* for the final EUROIA scale are displayed in Table 2.

**Table 2:** Psychometric properties of the EUROIA.

Domain	Items	Reliability (*α*)	Factor loading	Total variance explained (%)
**SOC-AFF**	Q13: I got together with my partner to be sexually intimate.	0.83	0.78	7.8
	Q7: I did an activity with my partner to spend time with them.	0.82	0.72	
	Q15: I got together with someone I care about, just to enjoy their company.	0.82	0.71	
**SOC-RR**	Q6: I gave up some of my time to help a person that needed support.	0.81	0.81	9.0
	Q5: I did an activity to support my family or friend.	0.82	0.78	
	Q16: I did something to help my family or friend stay safe or secure.	0.81	0.72	
**SLF-AFF**	Q18: I did an activity to feel calm and relaxed.	0.82	0.75	10.9
	Q14: I did something to help me feel more attractive.	0.82	0.64	
	Q17: I did something to take care of my well-being.	0.82	0.62	
	Q2: I did something special to feel part of a greater purpose.	0.82	0.51	
**EUD-WB**	Q9: I did a learning activity to help me master a skill.	0.81	0.77	31.5
	Q11: I took a course to improve my skill or knowledge.	0.82	0.74	
	Q8: I did something to feel connected to the world around me.	0.82	0.67	
	Q12: I did a special activity that gives me pleasure.	0.82	0.40	

Social Affiliation, SOC-AFF; Social Roles and Responsibilities, SOC-RR; Self-Affirmation, SLF-AFF; Eudaimonic Well-being, EUD-WB; Cronbach’s alpha, *α*

Mean scores and item distributions of the EUROIA by self-reported gender are available in Table 3. Men reported engaging in the following activities more frequently than women: Q7: “I did an activity with my partner to spend time with them”, Q9: “I did a learning activity to help me master a skill”, and Q13: “I got together with my partner to be sexually intimate”, while women reported greater frequency for Q14: “I did something to help me feel more attractive”.

**Table 3:** EUROIA scores and distributions by gender.

Instrument subscales	Men (*n* = 169)	Women (*n* = 114)	Total (*N* = 283)	*p-*value
**Q2: I did something special to feel part of a greater purpose.**				
Mean (SD)	2.2 (1.2)	2.3 (1.2)	2.2 (1.2)	0.31
Range	1.0, 4.0	1.0, 4.0	1.0, 4.0	
**Q5: I did an activity to support my family or friend.**				
Mean (SD)	2.6 (1.1)	2.7 (1.1)	2.7 (1.1)	0.74
Range	1.0, 4.0	1.0, 4.0	1.0, 4.0	
**Q16: I did something to help my family or friend stay safe or secure.**				
Mean (SD)	2.0 (1.0)	2.1 (1.0)	2.0 (1.0)	0.38
Range	1.0, 4.0	1.0, 4.0	1.0, 4.0	
**Q7: I did an activity with my partner to spend time with them.**				
Mean (SD)	2.9 (1.0)	2.6 (1.0)	2.8 (1.0)	0.01*
Range	1.0, 4.0	1.0, 4.0	1.0, 4.0	
**Q8: I did something to feel connected to the world around me.**				
Mean (SD)	2.5 (1.1)	2.3 (0.9)	2.4 (1.0)	0.10
Range	1.0, 4.0	1.0, 4.0	1.0, 4.0	
**Q9: I did a learning activity to help me master a skill.**				
Mean (SD)	2.1 (1.1)	1.8 (1.0)	2.0 (1.0)	0.04*
Range	1.0, 4.0	1.0, 4.0	1.0, 4.0	
**Q11: I took a course to improve my skill or knowledge.**				
Mean (SD)	1.8 (1.0)	1.7 (0.9)	1.7 (1.0)	0.36
Range	1.0, 4.0	1.0, 4.0	1.0, 4.0	
**Q12: I did a special activity that gives me pleasure.**				
Mean (SD)	3.0 (1.0)	2.8 (1.0)	2.9 (1.0)	0.08
Range	1.0, 4.0	1.0, 4.0	1.0, 4.0	
**Q13: I got together with my partner to be sexually intimate.**				
Mean (SD)	2.2 (1.1)	1.8 (1.0)	2.1 (1.1)	< 0.001*
Range	1.0, 4.0	1.0, 4.0	1.0, 4.0	
**Q14: I did something to help me feel more attractive.**				
Mean (SD)	1.8 (0.9)	2.1 (0.9)	1.9 (0.9)	0.01*
Range	1.0, 4.0	1.0, 4.0	1.0, 4.0	
**Q15: I got together with someone I care about, just to enjoy their company.**				
Mean (SD)	2.3 (0.9)	2.2 (0.9)	2.3 (0.9)	0.63
Range	1.0, 4.0	1.0, 4.0	1.0, 4.0	
**Q16: I did something to help my family or friend stay safe or secure.**				
Mean (SD)	2.5 (1.1)	2.5 (1.1)	2.5 (1.1)	1.00
Range	1.0, 4.0	1.0, 4.0	1.0, 4.0	
**Q17: I did something to take care of my well-being.**				
Mean (SD)	2.7 (1.1)	2.7 (1.2)	2.7 (1.1)	0.79
Range	1.0, 4.0	1.0, 4.0	1.0, 4.0	
**Q18: I did an activity to feel calm and relaxed.**				
Mean (SD)	2.2 (1.1)	2.4 (1.1)	2.3 (1.1)	0.10
Range	1.0, 4.0	1.0, 4.0	1.0, 4.0	

*p < 0.05

Standard deviation, SD

### CFA findings

**Fig 1:**
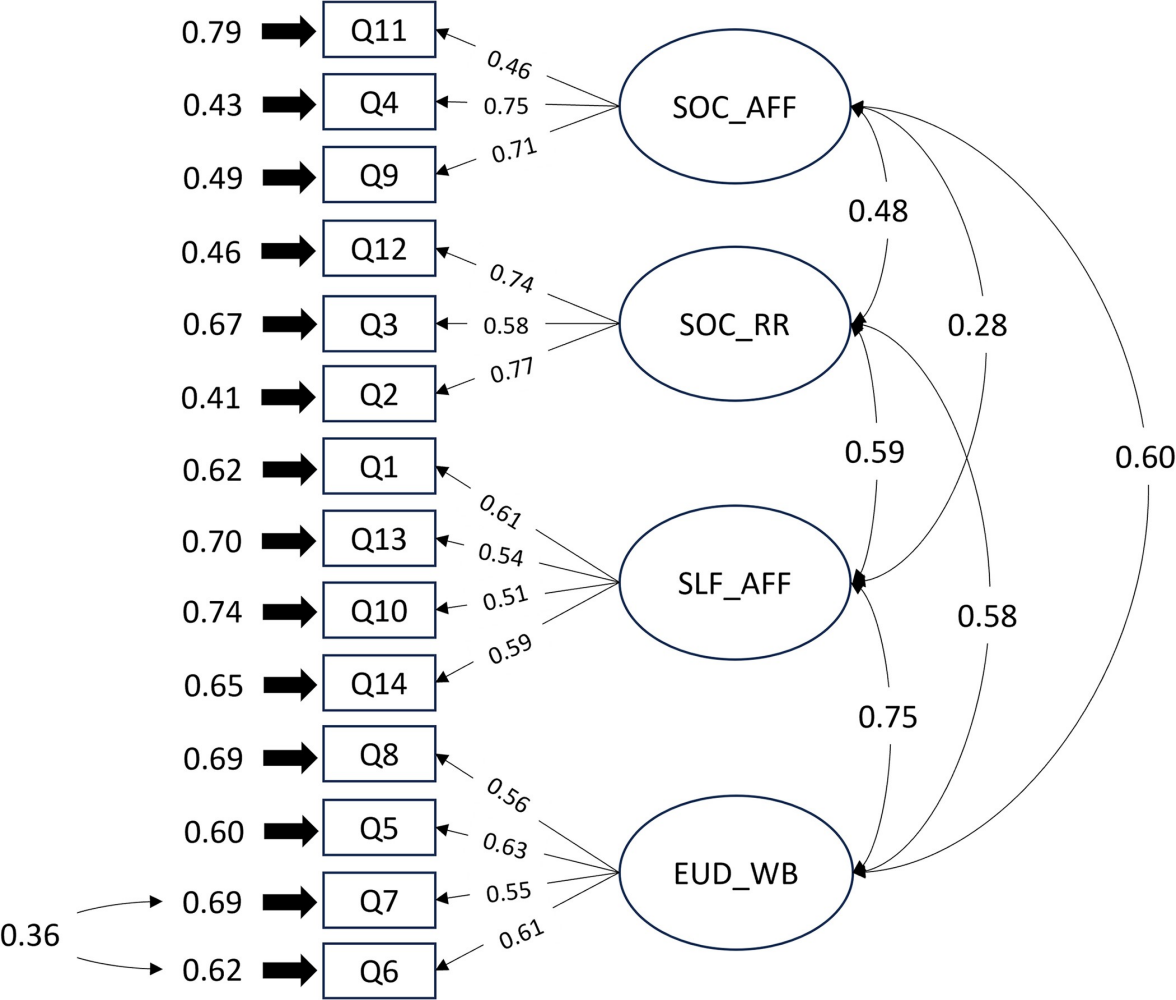
Model of the 14-item EUROIA with standardized factor loadings and correlations. Social Affiliation, SOC-AFF; Social Roles and Responsibilities, SOC-RR; Self-Affirmation, SLF-AFF; Eudaimonic Well-being, EUD-WB.

Following the PCA results, two 4-factor, first-order confirmatory models were tested based on the 14-item EUROIA. The collected data from our sample violated the normality assumption as estimated by Mardia’s multivariate normality test, therefore a maximum likelihood estimation with robust standard errors and SB scaled test statistic were used [46–48]. The results from our CFA are presented in Table 4 which provides a summary of the robust fit indices for both models. The baseline model (Model A) had sufficient global model fit (*χ*^2^ or df = 2.03; TLI = 0.88; CFI = 0.90; SRMR = 0.07; RMSEA = 0.07). However, local areas of strain represented by high modification indices (> 25) were present. After re-specifying Model A and applying residual correlations to Q9 and Q11, we obtained adequate global model fit with Model B (*χ*^2^ or df = 1.80; TLI = 0.90; CFI = 0.93; SRMR = 0.06; RMSEA = 0.06). Models were compared in likelihood ratio tests (Model B versus A), with B performing significantly better than A (ΔSB*χ*^2^ = 17.71, *p* < 0.001). Fig 1 outlines the final CFA of Model B with standardized factor loadings.

**Table 4:** Summary of robust fit statistics of CFA models. 1 residual correlation was applied between EQ9 (Master skill) and EQ11 (Improve skill).

	*χ*^2^ / df	TLI	CFI	SRMR	RMSEA (90% CI)	*χ*^2^ test of models
**Model A**	2.03	0.88	0.90	0.07	0.07 (0.05, 0.08)	Model B compared with Model A ΔSB*χ*^2^ = 17.71 *p* < 0.001
**Model B** 1 residual correlation	1.80	0.90	0.93	0.06	0.06 (0.04, 0.08)	

Tucker-Lewis Index, TLI; Comparative Fit Index, CFI; standardized root mean square residual, SRMR; root mean square error of approximation, RMSEA; degrees of freedom, df

The factor loading and proportions of variance explained by each item and factor are presented in Table 5, with all items having a factor loading greater than 0.40 and *p* < 0.001. Items had an R^2^ ranking from 0.21 to 0.59, which are acceptable as they are greater than 0.1. Composite reliability indicated good internal consistency for the overall scale (*Σ* = 0.89).

**Table 5:** Final factor loadings of Model B and proportion of variance explained.

Dimension and item	Factor loadings*	Proportion of variance explained
**SOC-AFF**		
Q9: I got together with my partner to be sexually intimate.	0.71	0.51
Q4: I did an activity with my partner to spend time with them.	0.76	0.57
Q11: I got together with someone I care about, just to enjoy their company.	0.46	0.21
**SOC-RR**		
Q2: I did an activity to support my family or friend.	0.77	0.59
Q3: I gave up some of my time to help a person that needed support.	0.58	0.33
Q12: I did something to help my family or friend stay safe or secure.	0.74	0.54
**SLF-AFF**		
Q1: I did something special to feel part of a greater purpose.	0.62	0.38
Q14: I did an activity to feel calm and relaxed.	0.59	0.35
Q10: I did something to help me feel more attractive.	0.51	0.26
Q13: I did something to take care of my well-being.	0.54	0.30
**EUD-WB**		
Q6: I did a learning activity to help me master a skill.	0.61	0.38
Q7: I took a course to improve my skill or knowledge.	0.55	0.31
Q5: I did something to feel connected to the world around me.	0.63	0.40
Q8: I did a special activity that gives me pleasure.	0.56	0.31

*Significance denoted by *p* < 0.001

Social Affiliation, SOC-AFF; Social Roles and Responsibilities, SOC-RR; Self-Affirmation, SLF-AFF; Eudaimonic Well-being, EUD-WB

## Discussion

This study developed and tested a psychometric scale, the EUROIA, which provides a measure of goal-directed activities that represent a key feature of a novel process-based model of HRQL [8, 16]. Development of the scale was inspired by a meta-theoretical model on various aspects of goal-directed activities [8]. The EUROIA scale was reduced to 14 items using PCA and grouped into four factors: SOC-AFF, SOC-RR, SLF-AFF, and EUD-WB. From the initial instrument, a fifth factor (connecting with a greater purpose) consisting of four items was removed due to redundancies in the scale, based on cross-factor loading and theoretical considerations. The CFA of the EUROIA obtained acceptable global model fit indices and composite reliability which supported the 4-factor structure proposed by the PCA and meta-theoretical model.

Development of the EUROIA instrument was based on theoretical or philosophical considerations of self-reported well-being and qualitative research in which patients self-described HRQL as a process of learning to accept and adapt to life post-diagnosis that was characterized by dynamic changes in health status, loss of personal autonomy, and a persistent existential threat that was accompanied by the sense that life was tenuous [8]. The process-based EUROIA instrument is grounded in clinical findings which suggest that individuals maintain or improve HRQL by initiating, monitoring, evaluating, and revising self-determined activities to promote well-being. This iterative process may be guided by (i) the self-management of illness-related stress and the utilization of available resources to optimize personal well-being [57, 58], (ii) the fulfillment of basic psychological needs for autonomy, relatedness, and competence [59], and (iii) the optimization of perceived self-efficacy in managing person-environment interactions. While state-based models of HRQL are well-established in providing clinically relevant descriptions of an individual’s HRQL status [60] and an estimate of risk of future events [61], it is unclear how they can be effectively used to inform patients on how to maintain or improve their HRQL [62]. The EUROIA’s process-based approach may provide an opportunity to inform and extend the design of interventions which promote adaptive adjustment to chronic illness, such as behavioural activation in cognitive behavioural therapy. There also exists a therapeutic potential to promote HRQL by guiding individuals to engage in activities that reflect personally meaningful life goals outside of a clinical context.

This project helps to extend clinically applied theory on HRQL and wellbeing. Alexandrova’s research on well-being [63] highlights a need to develop mid-level theories that specify how normative (abstract) theories of HRQL or well-being apply to the specific circumstances of an individual’s life. The latent variables identified in the present study reflect goal-directed activities that are pursued by individuals in the context of their daily life situations, as expressed along prototypical themes for eudaimonic wellbeing, fulfillment of social roles, self-affirmation, and social affiliation. The EUROIA is designed to establish a taxonomy of goal-directed activities that are used by individuals to optimize well-being and HRQL within the context of their daily life. This taxonomy is best established using self-report data obtained from a large sample of the general population, with representation across gender, ethno-racial identity, and socioeconomic status.

The present study acknowledges the potential limitations that may arise from the sample size (*N* = 275 participants), which includes limited sociodemographic characteristics. Many participants identified as ethno-racially White, educated men (having completed college or an undergraduate degree), with an annual household income of ≥ 100 000 CAD. These factors may restrict the generalizability of the study’s findings; however, our sample was defined a priori as those patients diagnosed with CHF or CKD, without severe co-morbidities, who had personal access to a computer and who self-reported as least basic competence in using a computer. Future research using the EUROIA will use stratified sampling to address generalizability. Our findings are not an exhaustive list of prototypical goal-directed activities for living well. Future research is needed to determine how EUROIA performs in different age groups, genders, and ethnic groups.

## Conclusion

The EUROIA’s 4-factor structure of HRQL was defined by PCA as activities that promote social affiliation, the fulfillment of social roles and responsibilities, self-affirmation, and eudaimonic well-being. This 14-item scale demonstrates satisfactory reliability and content validity, as well as good internal consistency. The EUROIA may be employed in clinical trials to provide informed feedback to patients on how they might maintain or modify their use of a specific category of goal-directed activities to optimize perceived well-being.

## References

[1] Centers for Disease Control and Prevention. Health-related quality of Llfe (HRQOL) [Available from: https://www.cdc.gov/hrqol/index.htm.

[2] Centers for Disease Control and Prevention. Health-related quality of life (HRQOL): Why is it important to track HRQOL? [Available from: https://www.cdc.gov/hrqol/concept.htm.

[3] Government of Canada. Measuring what matters: Toward a quality of life strategy for Canada. 2021.

[4] American Medical Association Journal of Ethics. Quality of life and clinical decision making [Available from: https://journalofethics.ama-assn.org/issue/quality-life-and-clinical-decision-making.

[5] European Medicines Agency. Regulatory guidance for the use of health-related quality of life (HRQL) measures in the evaluation of medicinal products—scientific guideline. 2005.

[6] Ware JE Jr, Sherbourne CD. The MOS 36-item short-form health survey (SF-36). I. Conceptual framework and item selection. Med Care. 1992;30(6):473–83. 1593914

[7] Taylor VR. Measuring healthy days: Population assessment of health-related quality of life. 2000.

[8] Nolan RP, & Sharpe M. J. A process-based approach to health-related quality of life as a “way of living”. Qual Life Res. 2023:1–10. 10.1007/s11136-023-03385-2 PMC10393893 37004629

[9] Brazier JE, Harper R., Jones N. M., O’Cathain A,. Thomas K. J., Usherwood T., et al. Validating the SF-36 health survey questionnaire: New outcome measure for primary care. BMJ. 1992;305(6846):160–4. doi: 10.1136/bmj.305.6846.160 1285753 PMC1883187

[10] Zullig KJ, Valois R. F., Huebner E. S., & Drane J. W. Evaluating the performance of the Centers for Disease Control and Prevention Core Health-Related Quality of Life Scale with adolescents. PHR. 2004;119(6):577–84. doi: 10.1016/j.phr.2004.09.007 15504449 PMC1497669

[11] Kielbergerová L, Mayer O., Vaněk J., Bruthans J., Wohlfahrt P., & Cífková R. Quality of life predictors in chronic stable post-stroke patients and prognostic value of SF-36 score as a mortality surrogate. Transl Stroke Res. 2015;6(5):375–83. doi: 10.1007/s12975-015-0418-6 26271301

[12] Hadot P. Philosophy as a way of life: Spiritual exercises from Socrates to Foucault: Blackwell; 1995.

[13] Hadot P. What is ancient philosophy? Cambridge, MA: Harvard University Press.

[14] Hadot P. The selected writings of Pierre Hadot: Philosophy as practice: Academic; 2020.

[15] Hadot P, Marcus A. The inner citadel: The meditations of Marcus AURELIUS: Harvard University Press; 1998.

[16] Syed F, Stogios N., Huszti E., Peiris R. G., & Nolan R. An assessment of goal-directed behaviours aimed at improving HRQL using the Evaluation of goal-directed behaviours to promote well being and health (EUROIA) Scale: A proof-of-concept study. Under review.

[17] Sprangers MAG, Schwartz CE. Integrating response shift into health-related quality of life research: a theoretical model. Social Science & Medicine. 1999;48(11):1507–15. doi: 10.1016/s0277-9536(99)00045-3 10400253

[18] Umberson D, Karas Montez J. Social Relationships and Health: A Flashpoint for Health Policy. Journal of Health and Social Behavior. 2010;51(1_suppl):S54–S66. 20943583 10.1177/0022146510383501 PMC3150158

[19] Kersten P, Borschel E, Neyer FJ, Mund M. The social side of personality: Do affiliation and intimacy motives moderate associations of personal relationships with well-being? Journal of Personality.n/a(n/a). doi: 10.1111/jopy.12746 35716149

[20] Greenaway KH, Cruwys T, Haslam SA, Jetten J. Social identities promote well-being because they satisfy global psychological needs. European Journal of Social Psychology. 2016;46(3):294–307.

[21] Prilleltensky I. Wellness as fairness. American journal of community psychology. 2012;49:1–21. doi: 10.1007/s10464-011-9448-8 21643926

[22] Prilleltensky I. Wellness without fairness: The missing link in psychology. Sage Publications Sage UK: London, England; 2013. p. 147–55.

[23] Duff J, Rubenstein C, Prilleltensky I. Wellness and fairness: Two core values for humanistic psychology. The Humanistic Psychologist. 2016;44(2):127.

[24] Di Martino S, Prilleltensky I. Happiness as fairness: The relationship between national life satisfaction and social justice in EU countries. Journal of community psychology. 2020;48(6):1997–2012. doi: 10.1002/jcop.22398 32627203

[25] Di Martino S, Scarpa MP, Prilleltensky I. Between wellness and fairness: The mediating role of autonomous human choice and social capital in OECD countries. Journal of community psychology. 2022;50(7):3156–80. doi: 10.1002/jcop.22822 35174508 PMC9544613

[26] Prilleltensky I, Scarpa MP, Ness O, Di Martino S. Mattering, wellness, and fairness: Psychosocial goods for the common good. American journal of orthopsychiatry. 2023. doi: 10.1037/ort0000668 37023268

[27] Emanuel AS, Howell JL, Taber JM, Ferrer RA, Klein WM, Harris PR. Spontaneous self-affirmation is associated with psychological well-being: Evidence from a US national adult survey sample. J Health Psychol. 2018;23(1):95–102. doi: 10.1177/1359105316643595 27160152

[28] Morgan J, Atkin L. Expelling Stress for Primary School Teachers: Self-Affirmation Increases Positive Emotions in Teaching and Emotion Reappraisal. International Journal of Environmental Research and Public Health. 2016;13(5):500. doi: 10.3390/ijerph13050500 27187437 PMC4881125

[29] Nelson SK, Fuller JA, Choi I, Lyubomirsky S. Beyond Self-Protection: Self-Affirmation Benefits Hedonic and Eudaimonic Well-Being. Pers Soc Psychol Bull. 2014;40(8):998–1011. doi: 10.1177/0146167214533389 24781897

[30] Main JL, Dillard AJ, editors. Effects of Self-affirmation on Coping and Motivational Systems 2012.

[31] Ryff CD, Singer BH. Know thyself and become what you are: A eudaimonic approach to psychological well-being. Journal of happiness studies. 2008;9:13–39.

[32] Ryff CD, Radler BT, Friedman EM. Persistent Psychological Well-being Predicts Improved Self-Rated Health Over 9–10 Years: Longitudinal Evidence from MIDUS. Health Psychol Open. 2015;2(2). doi: 10.1177/2055102915601582 26617988 PMC4662422

[33] Kaufman SB. Self-actualizing people in the 21st century: Integration with contemporary theory and research on personality and well-being. J Humanist Psychol. 2023;63(1):51–83.

[34] Ryff C. Self-realization and meaning-making in the face of adversity: A eudaimonic approach to human resilience. J Psychol Afr. 2014;24:1–12. doi: 10.1080/14330237.2014.904098 25435804 PMC4243302

[35] Kincaid JP, Fishburne R. P. Jr., Rogers R. L., Chissom B. S. Derivation of new readability formulas (automated readability index, fog count and flesch reading ease formula) for Navy-enlisted personnel. Naval Technical Training Command Millington TN Research Branch; 1975.

[36] Stull DE, Leidy NK, Parasuraman B, Chassany O. Optimal recall periods for patient-reported outcomes: challenges and potential solutions. Curr Med Res Opin. 2009;25(4):929–42. doi: 10.1185/03007990902774765 19257798

[37] ClinicalTrials.gov. ODYSSEE-vCHAT mental health program for heart failure and kidney disease patients https://clinicaltrials.gov/ct2/show/NCT05560737 [

[38] Peiris RG, Ross H., Chan C. T., Poon S., Auguste B. L., Rac V. E., et al. Automated digital counselling with social network support as a novel intervention for patients with heart failure: protocol for randomised controlled trial. BMJ Open. 2022;12(9):e059635. doi: 10.1136/bmjopen-2021-059635 36691152 PMC9445232

[39] Nolan RP, Ross HJ, Farkouh ME, Huszti E, Chan S, Toma M, et al. Automated E-Counseling for Chronic Heart Failure: CHF-CePPORT Trial. Circ Heart Fail. 2021;14(1):e007073. doi: 10.1161/CIRCHEARTFAILURE.120.007073 33464959

[40] General Assembly of the World Medical Association. World Medical Association Declaration of Helsinki: Ethical principles for medical research involving human subjects. JADA. 2014;81(3):14–8. 25951678

[41] R Core Team. R: A Language and Environment for Statistical Computing. Vienna, Austria: R Foundation for Statistical Computing; 2021.

[42] Revelle W. psych: Procedures for personality and psychological research. 2017.

[43] Rosseel Y. lavaan: An R package for structural equation modeling. J Stat Softw. 2012;48:1–36.

[44] O’Brien RM. Identification of simple measurement models with multiple latent variables and correlated errors. SM. 1994:137–70.

[45] Streiner DL, Norman G. R., & Cairney J. Health measurement scales: A practical guide to their development and use. New York: Oxford University Press; 2015.

[46] Kankainen A, Taskinen S., & Oja H. On Mardia’s tests of multinormality. Theory and Applications of Recent Robust Methods. 2004:153–64.

[47] Flora DB, & Flake J. K. The purpose and practice of exploratory and confirmatory factor analysis in psychological research: Decisions for scale development and validation. Can J Behav Sci. 2017;49(2):78.

[48] Li CH. The performance of ML, DWLS, and ULS estimation with robust corrections in structural equation models with ordinal variables. Psychol Methods. 2016;21(3):369–87. doi: 10.1037/met0000093 27571021

[49] Hu L, & Bentler P. M. Cutoff criteria for fit indexes in covariance structure analysis: Conventional criteria versus new alternatives. Struct Equ Modeling. 1999;6(1):1–55.

[50] Hu L, & Bentler P. M. Fit indices in covariance structure modeling: Sensitivity to underparameterized model misspecification. Psychol Methods. 1998;3(4):424.

[51] Cangur S, & Ercan I. Comparison of model fit indices used in structural equation modeling under multivariate normality. JMASM. 2015;14(1):14.

[52] Brown TA. Confirmatory factor analysis for applied research. New York: Guilford Publications; 2015.

[53] Gerbing DW, & Anderson J. C. On the meaning of within-factor correlated measurement errors. J Consum Res. 1984;11(1):572–80.

[54] Byrne BM. Factor analytic models: Viewing the structure of an assessment instrument from three perspectives. JPA. 2005;85(1):17–32. doi: 10.1207/s15327752jpa8501_02 16083381

[55] Whittaker TA. Using the modification index and standardized expected parameter change for model modification. J Exp Educ. 2012;80(1):26–44.

[56] Fu Y, Wen Z., & Wang Y. A comparison of reliability estimation based on confirmatory factor analysis and exploratory structural equation models. Educ Psychol Meas. 2022;82(2):205–24. doi: 10.1177/00131644211008953 35185157 PMC8850766

[57] Lazarus RS, Folkman S. Stress, appraisal, and coping. New York: Springer Pub. Co. New York; 1984. Available from: http://www.dawsonera.com/depp/reader/protected/external/AbstractView/S9780826141927.

[58] Leventhal H, Cahn S. Quality of life: A process view. Psychology and Health. 1997;12:753–67.

[59] Ryan RM, Deci EL. Self-determination theory: Basic psychological needs in motivation, development, and wellness. United States of America: Guilford Press; 2017. 1–756 p.

[60] Dominick KL, Ahern FM, Gold CH, Heller DA. Relationship of health-related quality of life to health care utilization and mortality among older adults. Aging Clin Exp Res. 2002;14(6):499–508. doi: 10.1007/BF03327351 12674491

[61] DeSalvo KB, Bloser N, Reynolds K, He J, Muntner P. Mortality prediction with a single general self-rated health question. A meta-analysis. J Gen Intern Med. 2006;21(3):267–75. doi: 10.1111/j.1525-1497.2005.00291.x 16336622 PMC1828094

[62] Greenhalgh J, Long AF, Flynn R. The use of patient reported outcome measures in routine clinical practice: lack of impact or lack of theory? Social Science & Medicine. 2005;60(4):833–43. doi: 10.1016/j.socscimed.2004.06.022 15571900

[63] Alexandrova A. A philosophy for the science of well-being. New York, NY: Oxford University Press; 2017. xlv, 196 pages p.

